# Sirolimus in the treatment of kaposiform lymphangiomatosis

**DOI:** 10.1186/s13023-021-01893-3

**Published:** 2021-06-08

**Authors:** Jiangyuan Zhou, Kaiying Yang, Siyuan Chen, Yi Ji

**Affiliations:** 1 Division of Oncology, Department of Pediatric Surgery, West China Hospital of Sichuan University, #37# Guo-Xue-Xiang, Chengdu, 610041 China; 2 Pediatric Intensive Care Unit, Department of Critical Care Medicine, West China Hospital of Sichuan University, #37# Guo-Xue-Xiang, Chengdu, 610041 China

**Keywords:** Vascular anomaly, Lymphatic malformation, Kaposiform lymphangiomatosis, Sirolimus, Mammalian target of rapamycin

## Abstract

**Background:**

Kaposiform lymphangiomatosis (KLA), which is a new subtype of generalized lymphatic anomaly, is a rare disease with a poor prognosis. Currently, there is no standard treatment due to the poor understanding of KLA. Sirolimus, which is an inhibitor of mammalian target of rapamycin, has been shown to have promising potential in the treatment of complicated vascular anomalies. The aim of this study was to introduce the use of sirolimus for the treatment of KLA and to highlight the challenges of managing this refractory disease.

**Results:**

We reported seven patients with KLA who received sirolimus therapy in our center. Combined with previously reported cases, 58.3% achieved a partial response, 25.0% had stable disease, and 16.7% experienced disease progression. No severe sirolimus-related adverse events occurred during treatment.

**Conclusions:**

This study suggests that sirolimus is currently an option for the treatment of KLA, and it is hoped that more specific therapies will be developed in the future. Rapid advances in basic science and clinical practice may facilitate the development of important new treatments for KLA.

**Supplementary Information:**

The online version contains supplementary material available at 10.1186/s13023-021-01893-3.

## Background

Kaposiform lymphangiomatosis (KLA) is a rare, infiltrative, multifocal or defused lymphatic anomaly that is classified as a new subtype of generalized lymphatic anomaly (GLA) [1]. It usually presents in children with hemorrhagic effusions and respiratory symptoms. Generally, the characteristic dispersed spindled lymphatic endothelial cells are arranged in sheets or clusters with malformed lymphatic vessels that can be differentiated from GLA and kaposiform hemangioendothelioma [2]. However, compared with GLA, KLA has an unfavorable prognosis, and the overall survival rate is approximately 34% [1, 3].

Because the mechanism of KLA remains largely unknown, the management of patients with KLA is clinically challenging. To date, no standard approach has been developed for the treatment of KLA. Surgical therapies, including resection, drainage, pleurodesis and ligation of the thoracic duct, are palliative [1]. Pharmacotherapies for complicated vascular anomalies have been investigated previously, including interferon-alpha, corticosteroids, and vincristine, but the responses to treatment have been varied and unpredictable [1, 4]. Meanwhile, the adverse neurological effects of interferon and vincristine and the effects of long-term corticosteroid use on growth have limited their use in clinical practice [5, 6]. Recent studies have identified sirolimus as a potential treatment for complicated vascular anomalies with good tolerability [7–9]. Some investigators believe that the combination of sirolimus with steroids and/or vincristine is effective at treating KLA [4]. However, most of the data were derived from case reports [9]. Herein, we report a series of patients with KLA in our center who were treated with sirolimus and review reported KLA patients treated with sirolimus from the literature [7–13], with the aim of highlighting the role of sirolimus in KLA and the challenges of managing this refractory disease.

## Methods

We performed a retrospective analysis of patients with KLA who presented to our hospital. The institutional review board of West China Hospital of Sichuan University approved this study. All procedures followed the relevant guidelines and regulations. The parents of all patients provided written informed consent. The diagnosis of KLA was established in our hospital on the basis of clinical presentation, imaging data, and histopathology. Some patients were included in our previous study [2]. Patients with KLA who were not treated with sirolimus or lacked sufficient data were excluded. Data collected from medical records included clinical characteristics, treatments and outcomes.

Moreover, considering the rarity of KLA and the small number of patients in our center, we conducted a literature review of the reported KLA cases treated with sirolimus on PubMed and Medline to better understand the role of sirolimus in the treatment of KLA.

Referring to the published literature [7] and taking into account the characteristics of KLA, we defined the treatment response as follows. Progressive disease (PD) was defined as a ≥ 20% increase in target lesions; partial response (PR) was defined as a ≥ 20% and < 100% reduction in the target lesions; complete response (CR) was defined as a 100% reduction in the target lesions; and stable disease (SD) was defined as a < 20% increase and < 20% decrease in the volumes of the target lesions. All adverse events were evaluated using CTCAE V4.0.

## Results

### Presentation of the cases

#### Patient 1

**Fig. 1:**
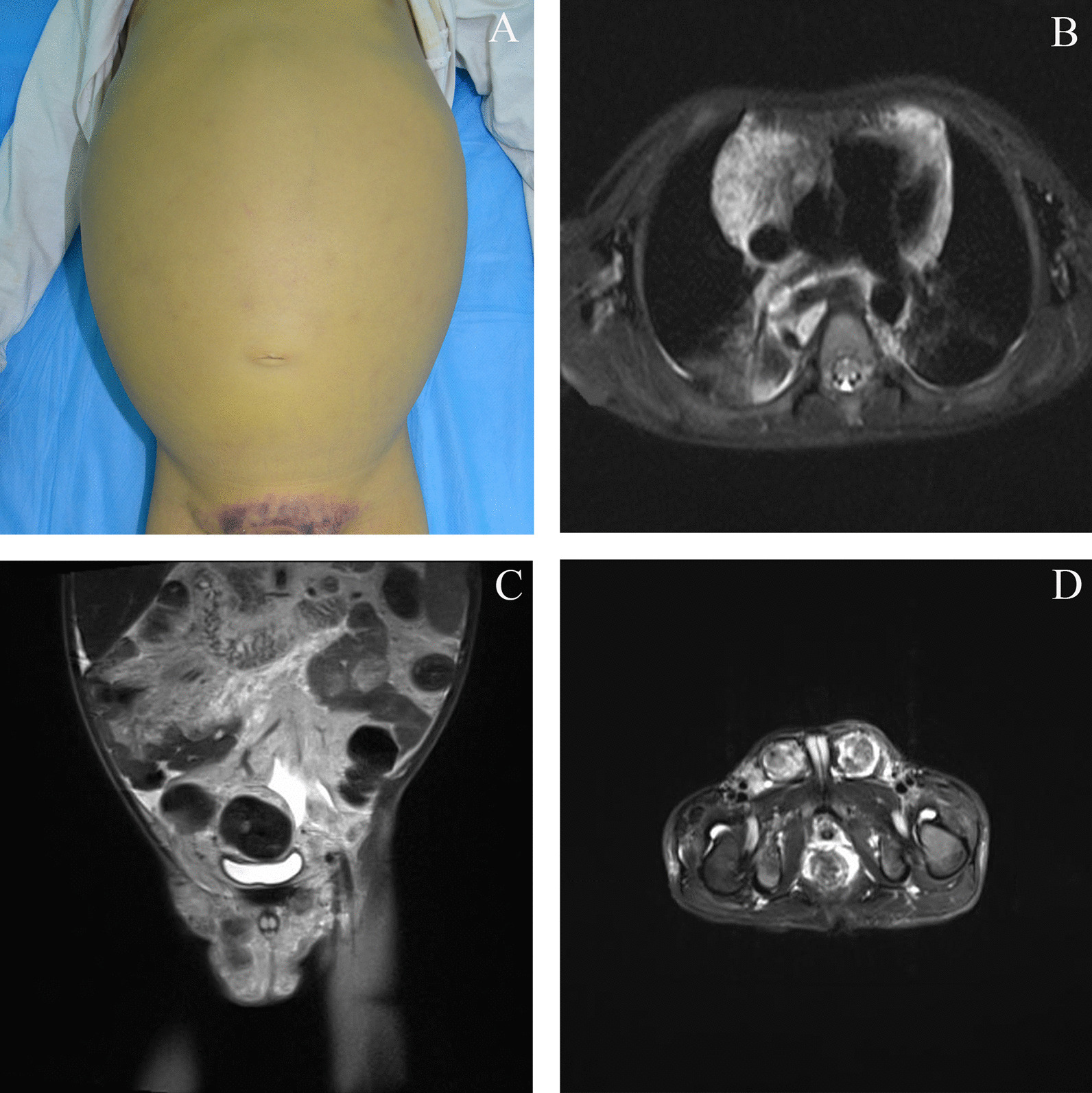
Patient 1 showed a grossly distended abdomen and perineal ecchymosis (**A**). Axial T2 fat-saturated image showed extensive mediastinal involvement (**B**). Coronal T2 fat-saturated image showed extensive abdominal cavity and pelvic cavity involvement (**C**). Axial T2 fat-saturated image showed bilateral inguinal regions, including testis involvement (**D**)

A 6-year-old boy was admitted to our hospital because of a right testis mass, abdominal distention and intermittent pain. He had a 4-year history of perineal ecchymosis accompanied by recurrent thrombocytopenia and coagulation disorders. Before admission to our hospital, the patient had been treated with corticosteroids, immunoglobulin and platelet transfusion. Sequential examinations demonstrated persistent consumptive coagulopathy and thrombocytopenia. Five months prior to referral to our hospital, he developed abdominal distention, intermittent pain and jaundice. Laboratory tests revealed that the platelet count was 10,000/μL, and fibrinogen was 0.49 g/L. Magnetic resonance imaging (MRI) revealed extensive heterogeneous enhanced lesions involving the mediastinum, abdominal cavity, pelvic cavity and bilateral inguinal regions and testes (Fig. 1). The diagnosis of KLA was confirmed by biopsy (Additional file 1: Figure S1). A combination treatment with prednisone (2 mg/kg/d), sirolimus (initial dose of 0.8 mg/m^2^ twice daily) and ademetionine (1000 mg/d) was commenced. However, the patient suffered from progressive tachypnea and abdominal distention even with active treatment. The boy died of multiple system organ failure after 2 months.

#### Patient 2

**Fig. 2:**
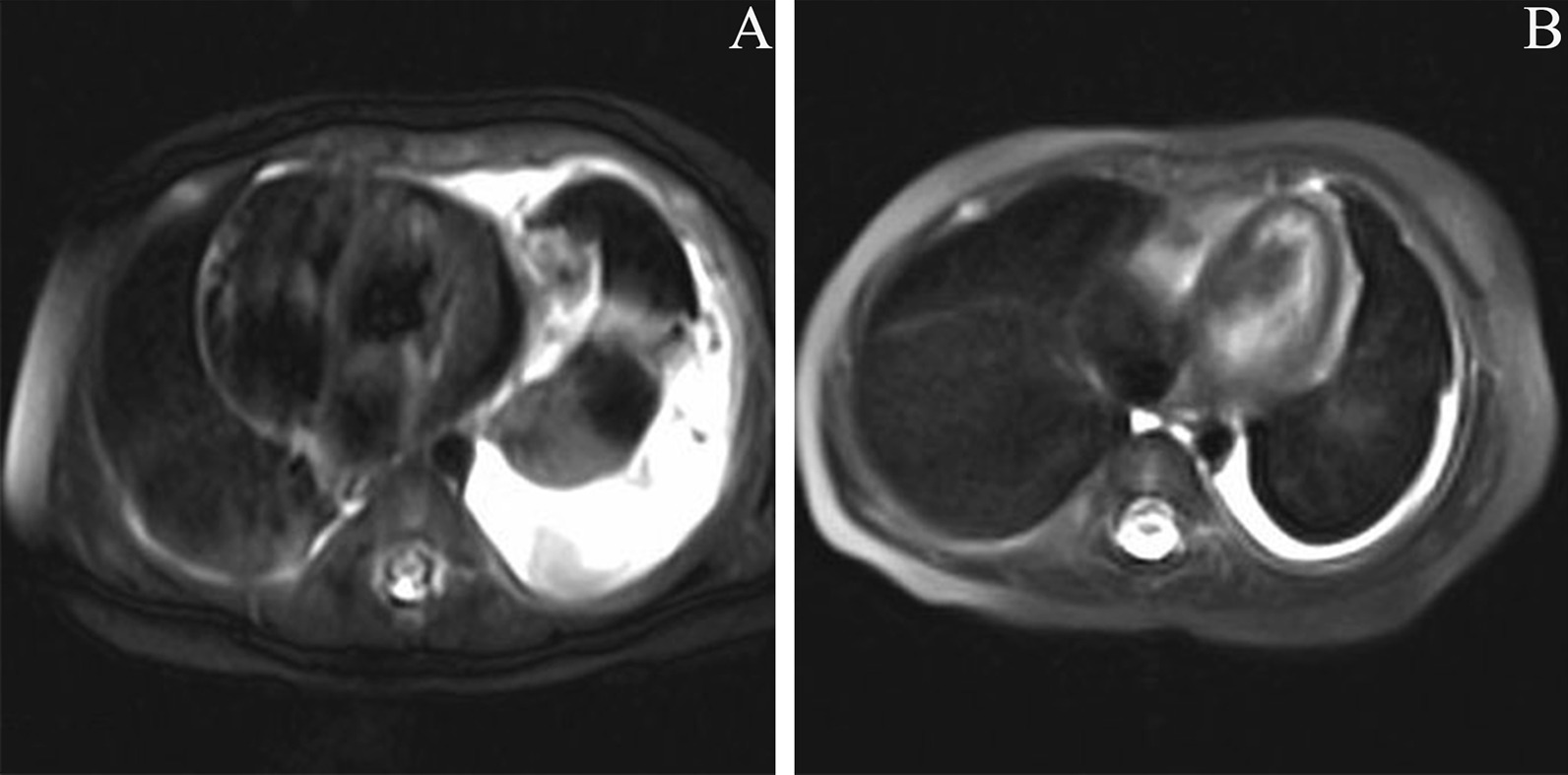
Patient 2 presented to our hospital because of dyspnea, shortness of breath, thrombocytopenia, and coagulopathy. T2-weighted MRI of the chest showed severe pleural effusion and thoracic lesions (**A**). After 5 months of treatment, thoracic MRI indicated a significant decrease in pleural effusion and a reduction in lesion size (**B**)

A 4-month-old girl presented to our hospital because of dyspnea and shortness of breath for 1 month. Image examination indicated severe pleural effusion and heterogeneous enhanced lesions involving the left mediastinum and left lung. The platelet count was 23,000/μL, and fibrinogen was 0.41 g/L. Considering the severe respiratory symptoms of the patient, thoracic drainage was performed. The drained fluid was hemorrhagic, but her pleural effusion was difficult to control. The girl was diagnosed with KLA. Then, sirolimus therapy (initial dose of 0.8 mg/m^2^ twice daily) combined with prednisone (2 mg/kg/day) was initiated. During the treatment, the plasma concentration of sirolimus was maintained within the range of 5–15 ng/ml. The hematological parameters returned to normal 2 months later, and prednisone was tapered. After 3 months of sirolimus treatment, her respiratory symptoms disappeared. Repeated MRI showed that the thoracic lesions shrunk and pleural effusion decreased significantly after 5 months of sirolimus therapy (Fig. 2). Two self-healing oral mucositis lesions occurred during sirolimus treatment. The patient is currently being treated with sirolimus monotherapy.

#### Patient 3

A 5-year-old boy was admitted to our hospital with dyspnea. Image examination suggested massive pleural effusion, and heterogeneous enhanced lesions involving both lungs, mediastinum, and multiple osteolytic lesions were found in vertebral bodies and ribs. The platelet count was 46,000/μL, and fibrinogen was 0.33 g/L. Thoracic drainage and fibrinogen transfusion were conducted to control the symptoms. The diagnosis of KLA was considered clinically and confirmed by biopsy. Then, sirolimus (initial dose of 0.8 mg/m^2^ twice daily) combined with corticosteroids (2 mg/kg/d) was administered. After 5 months of combined treatment, coagulation function improved, respiratory symptoms disappeared, and corticosteroids tapered. A partial response was achieved with 12 months of sirolimus treatment. He presented with slightly increased liver enzyme levels, which returned to normal after adjustment for the sirolimus dose. The patient is still being treated with sirolimus.

#### Patient 4

A 6-month-old boy was admitted to the hospital for abdominal distention and vomiting. MRI showed the extent of his lesions, including the lungs, mediastinum, spleen, liver, pancreas and mesentery. Laboratory tests revealed abnormal coagulation. A biopsy was performed and KLA confirmed. Aspirin (5 mg/kg/d) and vincristine (0.03 mg/kg once a week) plus corticosteroids (2 mg/kg/d) were administered successively, but the disease continued to progress. Considering the promising potential in treating complicated vascular anomalies, sirolimus (initial dose of 0.8 mg/m^2^ twice daily) was administered, and the lesions were stable, but the coagulation disorders did not improve until the use of corticosteroids (2 mg/kg/d). Six month later, corticosteroids tapered. No sirolimus-related adverse events occurred during treatment. To date, he has been treated with sirolimus for 41 months and has stable disease.

#### Patient 5

A 14-month-old boy was admitted to the hospital with complaints of cough and unexplained severe coagulopathy. Further examination found pleural and pericardial effusion and extensive lesions of the lungs and spleen. Pericardiocentesis and platelet transfusion were performed to control symptoms. Coagulation function improved, but respiratory function gradually deteriorated after splenectomy. At the same time, the diagnosis of KLA was established. Sirolimus (initial dose of 0.8 mg/m^2^ twice daily) monotherapy was administered, and the lesions of the lungs shrunk with symptoms ameliorated after 6 months of treatment, suggesting that sirolimus was effective in this case. He experienced no sirolimus-related adverse events during the treatment period. He is still being treated with sirolimus.

#### Patient 6

This 4-year-old boy presented with cough and was found to have left lung and mediastinal lesions with coagulopathy. After multidisciplinary discussion, the diagnosis of KLA was confirmed. He was treated with vincristine, but he had no response. He was subsequently treated with sirolimus (initial dose of 0.8 mg/m^2^ twice daily) plus short-term (4 months) prednisone (2 mg/kg/d) and had disease stabilization during 4 years of follow-up. He experienced upper respiratory tract infection and self-healing oral mucositis. Currently, he is still undergoing sirolimus monotherapy.

#### Patient 7

**Fig. 3:**
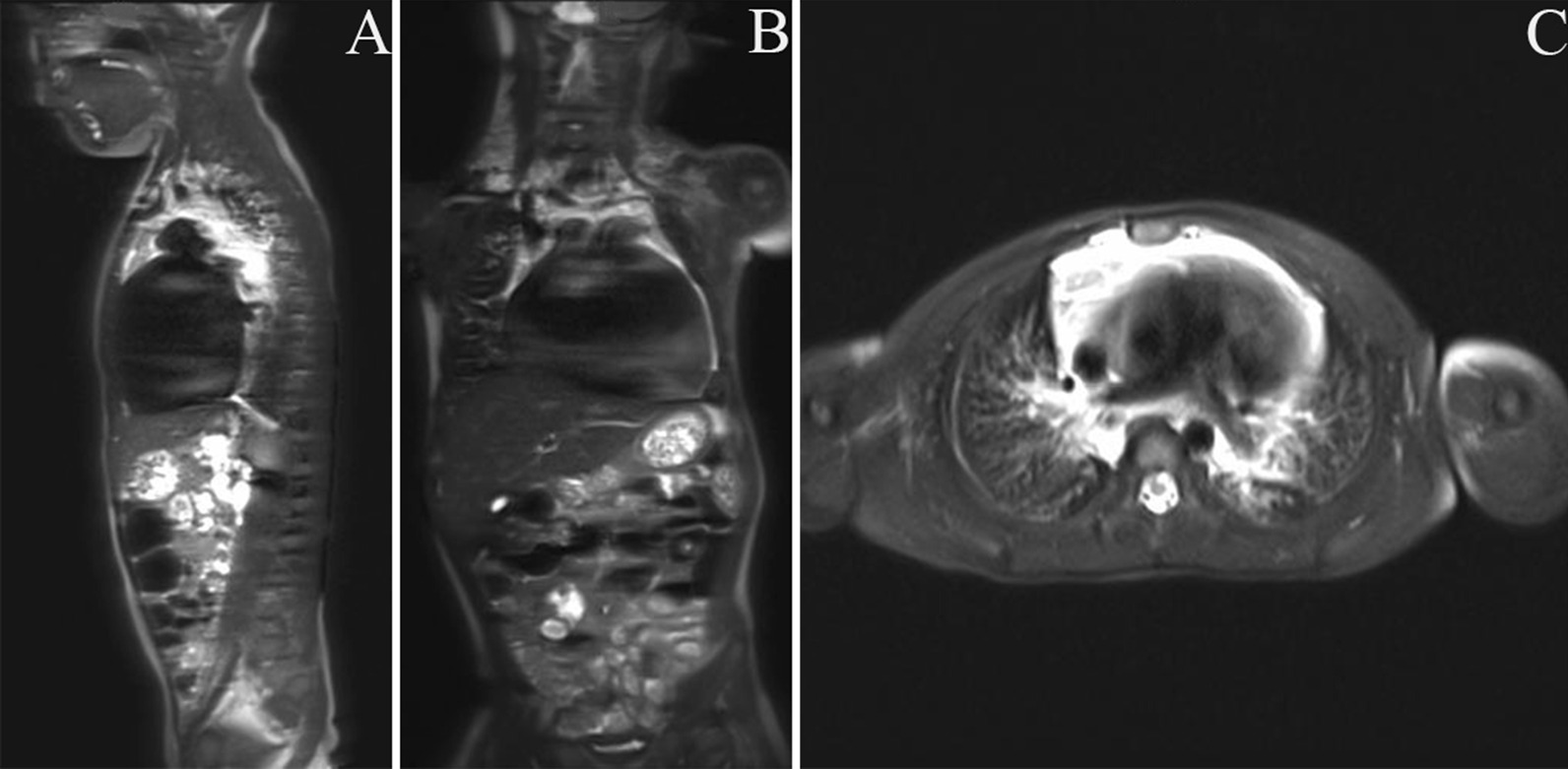
Patient 7 presented with dyspnea and cough. Imaging tests showed extensive lesions involving the mediastinum, lungs, neck and spleen

A 2-year-old boy presented with dyspnea and cough without fever, and laboratory examinations demonstrated unexplained coagulation disorders and thrombocytopenia. Initial imaging examination revealed massive pericardial effusion. Further MRI found extensive heterogeneous enhanced lesions involving the mediastinum, lungs, neck and spleen (Fig. 3). The diagnosis was changed from GLA to KLA by pathological examination. Treatment with sirolimus (initial dose of 0.8 mg/m^2^ twice daily), propranolol (2 mg/kg/d) and prednisone (2 mg/kg/d) was initiated and followed up regularly. No significant changes in the disease were seen during the first 6 months of treatment. At the seventh month of follow-up, coagulation function improved gradually, and platelets returned to normal. Then, propranolol and prednisone tapered. Currently, he is still being treated with sirolimus monotherapy, and his coagulation function is normal, while his foci lesions are stable.

The summary of all KLA patients is shown in Table 1.

**Table 1:** Summary of KLA patients

N	Age at start /sex	Location of target lesions	Major signs, symptoms, and/or complications	Previous treatments	Duration of sirolimus treatment (months)	Range (mean) of trough concentrations (ng/mL)	Response to sirolimus
1^a^	2 years/M	Mediastinum, abdominal cavity, pelvic cavity, bilateral inguinal regions and testes	Abdominal distention, pain and coagulation disorder	Corticosteroid, immune globulin and platelet transfusion	6	7.8–12.7 (10.4)	PD
2^a^	4 months/F	Left mediastinum and left lung	Dyspnea pleural effusion and coagulation disorder	Thoracic drainage	18	6.1–15.7 (10.2)	PR
3^a^	5 years/M	Lungs, vertebrae, ribs and mediastinum	Pleural effusion and coagulation disorder	Thoracic drainage, fibrinogen transfusion, exploratory thoracotomy	42	10.7–14.9 (13.3)	PR
4^a^	6 months/M	Lungs, mediastinum, spleen, liver, pancreas and mesentery	Abdominal distention, vomiting and coagulation disorder	Vincristine plus corticosteroid, aspirin	41	5.1–13.7 (11.6)	SD
5^a^	14 months/M	Lungs and spleen	Cough, respiratory distress, pleural effusion, pericardial effusion and coagulation disorder	Pericardiocentesis, corticosteroid, splenectomy and platelet transfusion	14	5.9–14.3 (12.1)	PR
6^a^	4 years/M	Left lung and mediastinum	Cough and coagulation disorder	Sirolimus, sirolimus plus steroids, sirolimus and vincristine	21	9.4–15.8 (10.6)	SD
7^a^	2 years/M	Mediastinum, lungs, neck and spleen	Dyspnea, cough and coagulation disorder	Propranolol and prednisone	21	10.0–14.7 (13.2)	SD
8^**b**^	8 years/M	Bone, thoracic and mediastinal	Chylothorax and coagulation disorder	Interferon and propranolol	30 (cessation)	4.4–9.0 (7.5)	PR
9^**b**^	8 years/M	Bone, thoracic and mediastinal	Scoliosis, chylothorax and coagulation disorder	Steroids and propranolol	24	8.1–12.4 (11.2)	SD
10^**b**^	20 years/M	Bone, thoracic and right chest wall	Gastrointestinal hemorrhage and coagulation disorder	Steroids	12	4.7–6.0 (5.5)	PR
11^**b**^	–/M	Thorax	NG	NG	NG	NG	PD
12^**b**^	7 years/M	Left scapular, left arm and thoracic vertebral bodies	Dyspnea, shortness of breath, pleural effusion and coagulation disorder	Chinese traditional medicine, pleurocentesis and albumin	8	9.3–13.4 (12.3)	PR
13^**b**^	NG	NG	NG	NG	12	10.0–15.0 (NG)	PR
14^**b**^	NG	NG	NG	NG	12	10.0–15.0 (NG)	PR
15^**b**^	NG	NG	NG	NG	12	10.0–15.0 (NG)	PR
16^**b**^	NG	NG	NG	NG	12	10.0–15.0 (NG)	PR
17^**b**^	NG	NG	NG	NG	12	10.0–15.0 (NG)	PR
18^**b**^	NG	NG	NG	NG	12	10.0–15.0 (NG)	PR
19^**b**^	NG	NG	NG	NG	12	10.0–15.0 (NG)	PD
20^**b**^	7 years/M	Abdominal cavity, lower neck, mediastinum, bone and left upper arm	Cephalohematoma and coagulation disorder	Steroids, vincristine and zoledronate	12	9.4–14.8 (NG)	PR
21^**b**^	13 years/F	Bone, mediastinum, spleen	Cough, fever, fatigue, intermittent wheezing, pleural effusion and coagulation disorder	Thoracostomy	3	NG	PR
22^**b**^	20 months/M	Mediastinum and spleen	Cough, pleural effusion, pericardial effusion, epidural hematoma and coagulation disorder	Diaphragmatic fenestrations	7	NG	PD
23^**b**^	11 years/F	Mediastinum, bone and spleen	Cough, chest pain, fatigue, listlessness, weight loss, coagulation disorder and pericardial effusion, coagulation disorder	Pericardiocentesis	31	NG	SD
24^**b**^	4 years/M	Bone, spleen and mediastinum	Fatigue, decreased appetite, weight loss, cough, spontaneous bruising, back pain, coagulation disorder and pleural effusion	Cryoprecipitate, platelet infusions and prednisolone	NG	NG	SD

*M* male, *F* female, *NG* not given, *PD* progressive disease, *PR* partial response, *SD* stable disease

^a^Data from our center

^**b**^Data from literature

## Discussion

Recently, KLA has been proposed as a new entity separate from GLA. However, although KLA has characteristics of malformations in histologic appearance, it has a more invasive nature [1, 4, 14]. The distinguishing features of KLA are intrathoracic lesions and hemorrhagic effusions [1]. KLA is multifocal or diffuse and usually involves the thoracic cavity, bone, retroperitoneum, abdominal viscera and soft tissues, which overlap with GLA [1, 15]. Because of its rarity, knowledge of KLA is extremely limited. In addition, the pathogenesis of KLA is not completely understood. Most recently, several studies detected the NRAS p. Q61R mutations in most patients with KLA [16, 17]. There is evidence suggesting that NRAS mutations are associated with cell proliferation through the MAPK and PI3K/AKT signaling pathways [18]. Similar to other vascular anomalies caused by mutations, NRAS somatic mutations may contribute to the occurrence of KLA.

Until now, no standard treatment regimen has been established due to the lack of firm evidence. Patients with KLA have poor outcomes despite aggressive management; the 5-year survival rate is 51%, and the overall survival rate is 34% [1]. Surgery is often impracticable due to its diffuse infiltrative nature, location and severe coagulation disorder [1]. Temporary improvement can be obtained via symptomatic treatments, but the lesions may rebound quickly [1]. Therefore, for most patients with KLA, pharmacologic interventions are an alternative treatment. Interferon-ɑ, corticosteroids, vincristine and sirolimus have been used to treat KLA in the past several years [1, 4]. Although there are various therapeutic approaches to KLA, the response is variable and suboptimal.

Sirolimus, which is an inhibitor of mammalian target of rapamycin (mTOR), plays a significant role in cell proliferation and migration through PI3K/AKT pathways. Recent studies have proven that sirolimus has promising potential in the treatment of complicated vascular anomalies [1, 7–9]. An in vitro experiment suggested that the PI3K/AKT and MAPK pathways are candidate therapeutic targets for KLA and that sirolimus could effectively inhibit KLA-derived cell proliferation [18]. Some experts recommend sirolimus combined with steroids and/or vincristine to treat KLA, but the relevant data are scarce.

In the present study, 3 of 7 patients partially responded to sirolimus. In these 4 patients, 3 patients obtained stable disease, and only 1 patient had progressive disease. Of the 24 reported patients with KLA treated with sirolimus [7–13], including 7 patients in our center, 14 (58.3%) achieved partial response, 6 (25.0%) showed stable disease, and only 4 (16.7%) experienced disease progression. Given the rarity of some diseases, such as KLA, repurposing existing drugs intended for the treatment of other conditions can be an option. Consistent with other studies, sirolimus was often administered after conventional treatments were ineffective. Notably, most KLA patients accepted combined therapy initially to control disease rather than sirolimus monotherapy. At the same time, the adverse events related to sirolimus during treatment in children suggest that sirolimus is well tolerated. For KLA patients, multidisciplinary collaborative supportive treatments are essential. Severe coagulopathy, pleural effusion and pericardial effusion require aggressive maneuvers to resolve them. Similar to kaposiform hemangioendothelioma, short-term combined therapy contributes to controlling the disease [19]. Later sirolimus monotherapy is sufficient. Considering the highly refractory nature of KLA, sirolimus is an optional medication at present.

However, no complete response was obtained in these patients, and only half of KLA patients achieved a partial response. Therefore, more attention needs to be given to this refractory disease. There is evidence suggesting that MEK inhibitors may be effective in patients with KLA after NRAS mutations are detected, but there are currently no clinical trials. Furthermore, trametinib has been reported to be effective in the treatment of KLA patients without NRAS mutations [20].

Early diagnosis is difficult in patients with KLA due to unspecific symptoms and lack of specific diagnostic indicators. Potential biomarkers such as angiogenic cytokines may be helpful in early diagnosis [21]. Therefore, patients with KLA who do not receive timely treatment may suffer poor outcomes. Furthermore, we believe that molecular diversity may contribute to the different effects of sirolimus and other targeted medicines, which was a limitation of this study. More studies should be conducted in the future to determine the progress of the abnormal development of lymphatic vessels and resolve current problems.

## Conclusions

In conclusion, initial short-term combined therapy and later long-term sirolimus monotherapy are currently optional strategies for the treatment of KLA, although their role is challenging in some patients. In the future, more studies are needed to better understand the pathophysiology of KLA, with the aim of supporting the development of more specific therapeutic approaches. It is likely that rapid advances in basic science and clinical practice will facilitate the development of important new approaches for the treatment of KLA.

## Supplementary Information

**Additional file 1**. Figure S1. The specimen of patient 1 was resected as a right testicular, which shows spindle lymphatic endothelial cells in clusters associated with malformed lymphatic channels (H&E staining, magnification × 200) (A). Immunostaining of anti-CD31 showed that the spindle cells were positive for CD31 (magnification × 100) (B). Immunostaining of anti-CD34 showed that the spindle cells were positive for CD34 (magnification × 100) (C). Immunostaining of D2-40 revealed proliferating lymphatic vessels (magnification × 100) (D).

## Data Availability

The datasets used and/or analyzed during the current study are available from the corresponding author upon reasonable request.

## Abbreviations

KLA: Kaposiform lymphangiomatosis
mTOR: Mammalian target of rapamycin
GLA: Generalized lymphatic anomaly
MRI: Magnetic resonance imaging
PD: Progressive disease
PR: Partial response
CR: Complete response
SD: Stable disease
