# Supplementary figures and images for: Sirolimus in the treatment of kaposiform lymphangiomatosis
Source: Orphanet J Rare Dis. 2021 Jun 8;16:260. doi: 10.1186/s13023-021-01893-3 (PMC8186093; doi:10.1186/s13023-021-01893-3)

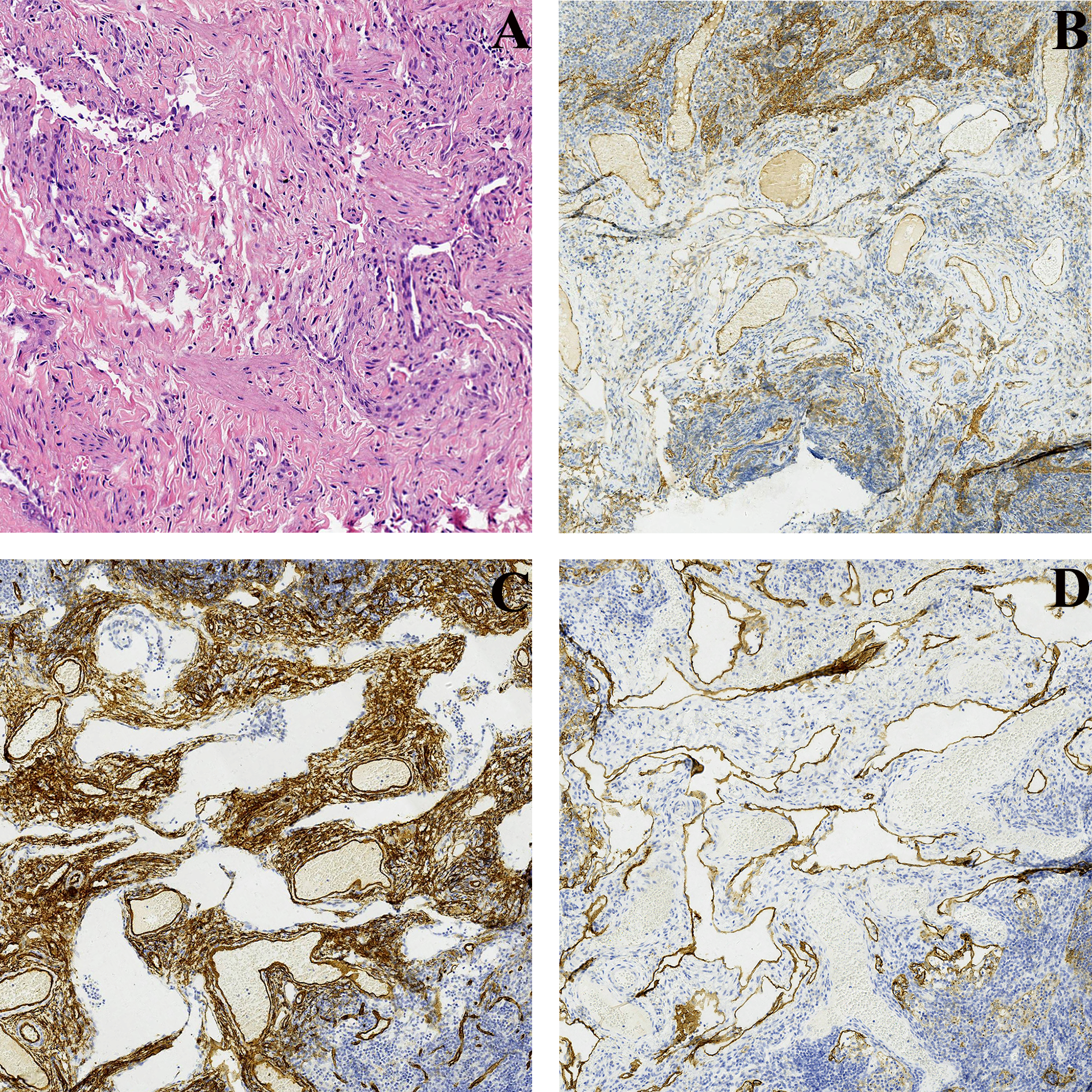

Supplement: Supplementary file 1 — Additional file 1. Figure S1. The specimen of patient 1 was resected as a right testicular, which shows spindle lymphatic endothelial cells in clusters associated with malformed lymphatic channels (H&E staining, magnification × 200) (A). Immunostaining of anti-CD31 showed that the spindle cells were positive for CD31 (magnification × 100) (B). Immunostaining of anti-CD34 showed that the spindle cells were positive for CD34 (magnification × 100) (C). Immunostaining of D2-40 revealed proliferating lymphatic vessels (magnification × 100) (D). [file 13023_2021_1893_MOESM1_ESM.tif]
